# Mixing Oil-Based Microencapsulation of Garlic Essential Oil: Impact of Incorporating Three Commercial Vegetable Oils on the Stability of Emulsions

**DOI:** 10.3390/foods10071637

**Published:** 2021-07-15

**Authors:** Yunjiao Zhao, Rui Liu, Cuiping Qi, Wen Li, Mohamed Rifky, Min Zhang, Ping Xiao, Tao Wu, Wenjie Sui

**Affiliations:** 1 State Key Laboratory of Food Nutrition and Safety, Tianjin University of Science & Technology, Tianjin 300457, China; zhaoyj9308@163.com (Y.Z.); q13821759620@163.com (C.Q.); 18335794579@163.com (W.L.); rifkyalm@esn.ac.lk (M.R.); wutaoxx@gmail.com (T.W.); wjsui@tust.edu.cn (W.S.); 2 College of Food Science and Bioengineering, Tianjin Agricultural University, Tianjin 300384, China; 3 Tianjin Chunfa Bio-Technology Group Co., Ltd., Tianjin 300300, China; xiaoping19860724@126.com

**Keywords:** garlic essential oil, emulsion stability, spray drying, microcapsule, vegetable oil

## Abstract

The active components in garlic essential oil are easily degradable, which limits its application in the food industry. Vegetable oils (VOs) were used to improve the stability of garlic essential oil (GEO) emulsion. The volatile compounds of GEO and its mixtures with vegetable oils (VOs), including corn oil (CO), soybean oil (SO), and olive oil (OO) indicated that GEO-VO mixtures had a higher percentage of Diallyl disulfide and Diallyl trisulfide than pure GEO. Adding an appropriate amount of VOs promoted the GEO emulsion (whey protein concentrate and inulin as the wall materials) stability in order of CO > SO > OO. Evaluation of the encapsulation efficiency, controlled release, and antimicrobial activity of GEO-VO microcapsules showed that the GEO was successfully entrapped and slowly released with active antibacterial activities on both *E. coli* and *S. aureus*. Collectively, these results implied that VOs, especially for 20% CO, improved the stability of GEO emulsions and the encapsulation efficiency of GEO microcapsules. The mechanism might be related to (1) the regulating effect of density difference between oil and water phases on prevention to gravitational separation, (2) the promotion to the compatibility of GEO and VOs to inhibit the phase separation caused by Ostwald ripening.

## 1. Introduction

Garlic (*Allium sativum* L.) is not only widely used around the world as a food and flavoring in cooking [1], but is also used as a traditional medicine against various human disorders and diseases, possessing antioxidant, antibacterial, anti-diabetic, anti-mutagenic and immunomodulation activities [2,3]. These functional properties can be ascribed partially to its volatile oil, which is mainly composed of a variety of sulfur-containing compounds, for instance, Diallyl sulfide, Diallyl disulfide, Diallyl trisulfide and 2-vinyl-4*H*-1,3-dithiin and others [4,5]. Currently, garlic essential oil (GEO) has been applied as an antibacterial, antioxidant agent and flavoring, particularly in several meat and chicken products [6]. However, the organosulfur-containing compounds in GEO are responsible for garlic’s unique aroma and flavor [7], and they are volatile and thermally unstable, thereby losing their functional properties when subjected to high temperature [8].

Microencapsulation is the most common technology used in the protection of essential oil, masking unpleasant orders and enhancing the solubility, including spray drying, coacervation, spray congealing, molecular inclusion, and so on [9]. Among them, spray drying is a highly promising microencapsulation technique for embedding GEO in the food industry because of its high flexibility, low economic costs, and continuously operating characteristics [10]. Several proteins (whey protein and soybean protein) and polysaccharides (maltodextrin, inulin, alginate, starch, and chitosan) have been used to microencapsulate essential oils [11,12]. Among them, whey protein is an abundant by-product of cheese production. It has been widely employed as the emulsifier and wall material in the encapsulation process due to its amphipathic groups. Fernandes et al. [13] reported that whey protein isolate (WPI)/inulin (IN) blends of 1:1 and 3:1 could effectively entrap rosemary essential oil with an encapsulation efficiency of 37.7% and 38.1%, respectively. Fernandes et al. [14] also employed WPI/IN to encapsulate ginger essential oil with the improved solubility and wettability properties of microcapsules as compared to pure WPI.

In addition to the selection of wall materials and the spray-drying operation parameters, the formation of a stable emulsion is an important factor that precedes the spray drying process of the GEO. The physicochemical nature of the occurrence of emulsion instability can be ascribed to different forms of mechanisms, such as gravitational separation, flocculation, coalescence, and phase inversion [15]. Therefore, research on the emulsion stability of spicy essential oil mainly focused on the selection and optimization of wall materials and the processing technology to enhance the emulsion dispersion and stability [4,8,16,17]. However, there are few studies on the improvement of the properties of spicy essential oil.

Accordingly, the objectives of the present work were to employ different vegetable oils (VOs) to alter the physicochemical properties of GEO. The impact of corn oil (CO), soybean oil (SO), and olive oil (OO) additions on the volatile compounds of GEO, the stability of GEO emulsions, and the encapsulation efficiency of GEO microcapsules was investigated at different concentrations of VOs. The controlled release and antibacterial properties of mixing VO-based GEO microcapsules were also studied to reveal the further effects of VOs addition on microencapsulation of GEO emulsions. This study is of great significance for improving the stability of GEO and stabilizing its flavoring composition, thus prolonging the shelf-life and widening GEO applications in the food industry.

## 2. Materials and Methods

### 2.1. Materials and Microorganisms

Garlic essential oil (GEO) was purchased from Taicheng Natural Fragrance Co., Ltd. (Ji’an, China). Whey protein concentrate (WPC) was composed of 80.3% protein, 5.1% water, 3.5% lactose, 3.4% starch, 3.8% fat, and 3.9% ash, which was purchased from New Zealand Fonterra Company (Auckland, New Zealand). Inulin (IN, >10 monomers, Beneo-Orafti^TM^ GR) was purchased from Beneo-Orafti (Tienen, Belgium). Maltodextrin (MD) was purchased from Xi’an Huibang Bioengineering Corporation (Xi’an, China). Vegetable oils, including corn oil (CO), soybean oil (SO), and olive oil (OO), were purchased from the local market in Tianjin, China. Gram-negative bacteria *Escherichia coli* (*E. coli*) ATCC 25922 and Gram-positive bacteria *Staphylococcus aureus* (*S. aureus*) ATCC 26112 were provided by the Culture Preservation Center of Tianjin University of Science & Technology. All other chemicals and reagents used were of analytical grade.

### 2.2. Volatile Compounds Analysis of GEO and GEO-Vegetable Oil Mixtures

Gas chromatography, coupled with mass spectrometry (GC–MS), was used to determine the impact of different vegetable oils (VOs) on the volatile compounds of GEO. Prior to analysis, GEO and GEO–vegetable oil (GEO-VO) mixtures (GEO:VO = 1:4) were placed in a 15 mL sample vial for solid-phase microextraction. The headspace solid-phase microextraction was performed to obtain the volatile compounds, referring to Lio et al. [18] and modified slightly. The extraction head was aged at 250 °C for 22 min, and inserted into the top space part of the sample vial. After extraction at 25 °C for 45 min, it was inserted into the injection port of GC–MS and performed thermal desorption for 25 min. According to the methods of Yong et al. [19], volatile compounds of GEO and GEO-VO mixtures were measured on a 7890B/7000C GC–MS system with an HP-5 MS fused silica capillary column (30 m × 0.25 mm, 0.25 μm film thickness) using helium as carrier gas at a constant pressure of 91.65 kPa and an average linear velocity of 22.693 cm/s. The injection temperature was 250 °C, and the split ratio was 10:1. The compounds eluting from the column were distributed to the detector of the mass spectrometer in EI mode at 70 eV with an ion source temperature of 230 °C and a mass scan range of 40–500 *m*/*z*. The volatile compounds of GEO and GEO-VO mixtures were identified by comparing their GC–MS information with the National Institute of Standards and Technology (NIST14) library data. Quantitative analysis was carried out by peak area normalization method and the content of each compound in the essential oil was expressed as area percentage.

### 2.3. Preparation of GEO and GEO-VO Emulsions

The formulation of wall materials (WPC/IN and WPC/MD) was optimized in terms of emulsion physical and flavor stability according to previous studies [14,20]. GEO emulsion had the highest physical stability and best flavor retention capacity when emulsified by WPC and IN at a ratio of 3:1 as wall materials (Supporting Information, Table S1, Figures S1 and S2). For the formulation of GEO and GEO-VO emulsions, briefly, the 20% (*w*/*v*) WPC-IN solution (WPC/IN = 3:1) was prepared in distilled water and kept at 25 °C for 12 h to ensure complete hydration. GEO was added into WPC/IN solution (GEO:WPC/IN ratio = 1:4) and mixed with different addition amounts of CO, SO, OO (0%, 10%, 13%, 20%, 33% and 50% of GEO, *w*/*w*), respectively. The mixtures were homogenized separately with an Ultra-Turrax homogenizer (T25, IKA, Staufen im Breisgau, Germany) at 1000 r/min for 5 min to obtain the primary GEO and GEO-VO emulsions. The primary emulsions were immediately ultrasonic-treated on a JY92-2D Vibra-cell probe sonicator (Ningbo Xinzhi Biological Technology Co. Ltd., Ningbo, China) in a pulse mode for 2 min (5 s ON and 5 s OFF) at 20 kHz and 160 W to produce GEO and GEO-VO emulsions, according to the method of Vall-llosera, et al. [21].

### 2.4. Characterizations of GEO and GEO-VO Emulsions

#### 2.4.1. Droplet Size Distribution Measurement

The droplet size distribution was measured using a BT-9300S laser analyzer (Dandong Bettersize Instruments Ltd., Dandong, China) with 0.1% sodium dodecyl sulfate as a dispersion medium at 25 °C.

#### 2.4.2. Determination of Turbiscan Stability Index

The physical stability was measured with Turbiscan ASG (Formulaction, Toulouse, France), which simultaneously records light transmission and backscattering on the dispersed oil droplets. The analyzed emulsion (20 mL) was put into a flat-bottomed cylindrical glass vial of 14 cm height and 16 mm diameter and then placed in the thermostatic measurement chamber. Scans were carried out hourly at 25 °C for 12 h, and the measurements were repeated three times independently. Turbiscan stability index (TSI) was calculated according to Wiśniewska, et al. [22].

#### 2.4.3. Confocal Laser Scanning Microscopy

The emulsion microstructure was observed using a Nikon Ti-U confocal laser scanning microscopy (CLSM). Nile red and fluorescein isothiocyanate (FITC) were used to visualize oil droplets and proteins, respectively. Briefly, emulsions (2 μL) were thoroughly mixed with 20 μL of Nile red solution (0.1%, *w*/*v*) and 20 μL FITC solution (0.05%, *w*/*v*), and covered with a cover glass. Samples were illuminated with the argon-ion laser at 488 nm and helium–neon laser at 543 nm. The image resolution amounted to 1024 pixels × 1024 pixels.

### 2.5. Preparation of GEO and GEO-VO Microcapsules

Spray drying was performed using a YC-015 spray dryer (Yacheng Instrument Equipment Co., Ltd., Shanghai, China) with an inlet air temperature of 170 °C and outlet air temperature of 60 °C. The obtained microencapsulated GEO and GEO-VO powders were sealed and stored in a dark environment at 4 °C for further analysis.

### 2.6. Characterizations of GEO and GEO-VO Microcapsules

#### 2.6.1. Encapsulation Efficiency

Encapsulation efficiency was determined according to the method of Wu et al. [23]. Briefly, the microencapsulated powder (1 g) was immersed in 20 mL of distilled water. The mixture was homogenized with IKA Ultra-Turrax homogenizer for 1 min, then mixed with 10 mL of *n*-hexane and stirred at 45 °C for 30 min, followed by centrifuging at 3000 r/min for 5 min. The supernatant was transferred to a 50 mL volumetric flask, and the sediment was subjected to the extraction-centrifugation process repeatedly four times. Five batches of supernatants were collected and diluted to 50 mL by *n*-hexane. The amount of GEO was determined by UV detection at 212 nm, according to the established calibration curve derived from GEO standards. The encapsulation efficiency (*EE*) of microcapsules was calculated using this equation:

(1) $$EE\left(\%\right)=\frac{M}{M_{0}}\times 100$$

where *M* is the mass of GEO loaded in the microcapsules, *M*_0_ is the total mass of GEO added.

#### 2.6.2. Fourier Transform Infrared (FTIR) Spectroscopy

GEO and GEO-VO microcapsules (1 mg) were ground with 150 mg KBr and then pressed into a pellet for FTIR analysis. FTIR measurements were performed using a Bruker FTIR spectrometer (model Vector 22) with a resolution of 4 cm^−1^ and an accumulation of 16 scans in the range of 4000–500 cm^−1^.

#### 2.6.3. Thermogravimetric Analysis

The thermal stability was evaluated upon thermogravimetric analysis (TGA) on a TGA-Q50 thermobalance (TA Instruments, Milford, MA, USA) under nitrogen atmosphere, with heating from 50 °C to 550 °C at a heating rate of 10 °C/min.

#### 2.6.4. Morphology and Size Distribution

Microcapsules were attached to a double-sided carbon tape adhered to a scanning electron microscope (SEM) stub, and coated with gold in a vacuum evaporator. The morphologies of microcapsules were observed using a Philips XL-3 scanning electron microscope (Philips Eindhoven, Amsterdam, The Netherlands) at an accelerating voltage of 20 kV, with a magnification of ×1000.

The particle size distribution was determined using a BT-9300S laser analyzer with ethanol as a dispersion medium at 25 °C.

### 2.7. Controlled Release

The controlled release of GEO from microcapsules was evaluated according to Campelo-Felix et al. [24], with some modifications. GEO and GEO-VO microcapsules (100 mg) were dispersed in 25 mL of *n*-hexane at flasks and placed in a shaker incubator at 30 °C with a rotation speed of 100 r/min. The samples were taken out at 0, 15, 30, 45, 60, 90, 120, 150, 180, and 240 min. The solution of each sample was centrifuged at 3000 r/min for 10 min and filtered through a 0.45-μm nylon membrane. The released GEO concentration was detected using a UV-Vis spectrophotometer at a wavelength of 212 nm. The GEO concentration standard curve was given as the following equation (*R*^2^ = 0.998):

(2) GEO concentration (mg/mL) = −0.00747 + 0.03805 × *Abs*

### 2.8. Antibacterial Activity

*E. coli* and *S. aureus* were employed to evaluate the antibacterial activities of GEO and GEO-VO microcapsules. Both bacteria were stored at −80 °C and refreshed on nutrient broth (NB)-agar media for growth. The activated bacteria were inoculated at 37 °C for 24 h and diluted into a bacteria concentration of 10^3^ CFU/mL. GEO and GEO-VO microcapsules (10 mg) were diluted with NB-agar media to create the desired gradient concentrations, adopting a 2-fold concentration gradient dilution method, then fully mixed with 1 mL of *E. coli* and *S. aureus* suspension. An aliquot of 200 μL of the suspension was placed in the 100-well honeycomb plate. The inhibition of the growth curve was measured using a Bioscreen C optical density-monitoring system (OY Growth Curves, Helsinki, Finland) at 37 °C for 48 h. The optical density (OD) value at 600 nm was recorded to estimate the inhibition effects of GEO and GEO-VO microcapsules on bacteria.

### 2.9. Statistical Analysis

The results are presented as mean ± standard deviation (SD) from three independent experiments. The data were analyzed by one-way analysis of variance (ANOVA), followed by Tukey’s HSD multiple comparison test. Differences were considered statistically significant at *p* < 0.05.

## 3. Results and Discussion

### 3.1. Volatile Compounds of GEO and GEO-VO Mixtures

The volatile compounds of GEO are influenced by the addition of different VOs (corn oil, soybean oil, and olive oil). The identification of the volatile-compound changes of GEO is critical to understand its functional properties, which are related partly to sulfur-containing compounds. The compounds in GEO and their % peak area were listed in Table 1. A total of 16 different volatile compounds were identified in GEO. Among them, 3-prop-2-enylsulfanylprop-1-ene (Diallyl sulfide) and 3-(prop-2-enyltrisulfanyl)prop-1-ene (Diallyl trisulfide) were the predominant compounds and accounted for 30.34% and 28.18%, respectively. Furthermore, 12 volatile compounds representing more than 1% comprised 98.56% of the total GEO volatile detected constituents. According to a previous study [25], a volatile compound accounting for above 5% of the total detected compounds can be regarded as a major chemical constituent. Five major sulfur compounds, namely Diallyl sulfide, 3-(prop-2-enyldisulfanyl)prop-1-ene (Diallyl disulfide), 4*H*-trithiine, 2-ethenyl-4*H*-1,3-dithiine, and Diallyl trisulfide accounted for 76.91% of the total GEO [26,27,28]. Satyal et al. [29] found that both garlic and wild garlic were dominated by allyl polysulfides. In addition, 9 sulfur compounds in GEO accounted for 90.18% of the total compounds’ content, which was inconsistent with the findings that the volatile sulfur compounds comprised 84.30–98.9% of the total GEO extracted by steam distillation method [4], and Diallyl disulfide and Diallyl trisulfide accounted for above 50% of the total volatile compounds in GEO [30]. There was no Diallyl disulfide or Diallyl trisulfide identified in corn oil, soybean oil, and olive oil (shown in Table S2). Similar sulfur compounds detected in GEO were also identified in GEO-CO, GEO-SO, and GEO-OO mixtures. Eight sulfur compounds were also identified in GEO-CO, GEO-SO, and GEO-OO, correspondingly comprising 96.53%, 96.74%, and 96.94% of the total GEO-VO, respectively. However, 2-prop-2-enylsulfanyl-1-(prop-2-enyltrisulfanyl) propane (Trisulfide, 2-propenyl 2-(2-propenylthio)propyl) was the only sulfur-containing compound that was not detected in GEO-VOs. Its relative content was as low as 0.38%, and it might be better solubilized in VOs, which led to the failure of detection when GEO mixed with VOs. The result indicated that no new flavor compounds were introduced and all-important sulfur-containing compounds were preserved by the addition of VOs, but the relative percentage of main sulfur compounds changed significantly, showing different compatibility of VOs and GEO.

### 3.2. Droplet Size Distributions of GEO Emulsions

In general, the emulsions are considered unstable due to Ostwald ripening, and small droplets tend to aggregate into large droplets, which may even cause the oil and water phases to separate [25]. Based on previous studies [14,31], the emulsion droplet size stabilized by whey protein and inulin/maltodextrin blends was ranging from a few microns to tens of microns. In the present study, the mean droplet size distributions of GEO and GEO-VO emulsions were influenced by the addition of VOs (Table 2 and Figure S4). For freshly prepared GEO-CO emulsion, the mean droplet size of GEO emulsions decreased with the increase of the CO addition amount, ranging from 1.31 μm without corn oil to 1.25 μm with 20% corn oil, and then increased with CO addition on the rise. A similar trend was observed after the GEO-CO emulsion was kept for 12 h. The droplet size and polydispersity index (PDI) value had minimum values of 1.95 μm and 2.67 by adding 20% CO. For GEO-SO and GEO-OO emulsions prepared freshly and kept for 12 h, the droplet sizes and PDI values also decreased first and then increased along with the VO additions rising. Both the freshly prepared GEO-SO emulsions and the emulsions after standing for 12 h had a minimum value of droplet sizes (1.28 μm and 2.07 μm) with the addition of 20% SO. While the droplet size of freshly prepared GEO-OO emulsions was smallest (1.20 μm) when adding 13% of OO, the minimum PDI value was obtained by adding 20% of OO. After 12 h of equilibration, the smallest droplet size and PDI value were achieved by adding 20% of OO. Thus, the GEO-OO emulsion with the addition of 13% OO was less stable than that mixed with 20% OO, indicating that the soluble components were rebalanced between different oil droplets [32]. However, the mean droplet size of GEO-OO emulsion with the addition of 20% OO was dramatically increased to 3.87 μm after remaining for 12 h. Therefore, based on the fluctuation of droplet size over time, it was implied that adding an appropriate amount of VOs could promote the stability of GEO emulsion to a certain extent, and the order of the stabilization effect of three VOs on GEO emulsion was as follows: CO > SO > OO.

The emulsions containing relatively high water-soluble flavor oil are considered unstable to droplet growth owing to Ostwald ripening, which is a process of growth of large droplets at the expense of the smaller ones and is adverse to the stability of the emulsion [33,34,35]. The water-soluble oil droplet trended to become larger, resulting in the larger droplets enriched with water-soluble oil and the smaller droplets enriched in water-insoluble oil. Studies indicated that mixtures of water-insoluble oil and essential oil could inhibit Ostwald ripening to some extent. As an inhibitor, water-insoluble oils are usually non-polar, high molecular weight substances, such as corn oil [36], sunflower seed oil [37] and medium-chain triglyceride [38]. Moreover, the size and length of hydrophilic group in emulsifiers, as well as emulsifier concentration, also affected the properties of interfacial film [39]. For instance, the droplet growth of orange-oil emulsion could be effectively inhibited by incorporating above 10% corn oil into the oil phase [32]. Therefore, the physical stability of GEO emulsion was enhanced by incorporating relatively high water-insoluble oil, which might be partially attributed to a suppressing mechanism of Ostwald ripening [40]. However, excessive addition of VOs (50%) accelerated the instability of the emulsion, resulting in a larger droplet size, which could not be clarified by the Ostwald ripening mechanism.

### 3.3. Turbiscan Stability Index of GEO Emulsions

The effects of VOs addition (0%, 10%, 13%, 20%, 33%, and 50%) on the Turbiscan stability index (TSI) of GEO emulsions were shown in Table 2. A smaller TSI value indicates a more stable emulsion [23]. For GEO-CO emulsions, TSI values of GEO emulsions incorporating 10%, 13%, and 20% CO were significantly lower than that of pure GEO emulsion, while the GEO emulsion with 50% CO addition had the highest TSI value, indicating that appropriately adding CO in the range of 10–20% could enhance the stability of GEO emulsion. For GEO-SO and GEO-OO emulsions, lower TSI values of GEO emulsions could be observed with the addition of 13–20% SO and 20% OO in comparison with that of pure GEO emulsion. As can be seen in Table 2, the three different GEO-VOs emulsions had the lowest TSI value at 20% VOs addition, and the TSI values of GEO-CO emulsions increased with storage time. After 12 h of storage, CO stabilized the GEO emulsion more effectively than SO and OO, as the TSI value was in an order of CO < SO < OO. Consistently with the droplet-size results, adding a certain proportion of VOs could partially improve the physical stability of GEO emulsions, but stabilization efficiency varied.

Abismail et al. [41] reported that the density difference between dispersed and continuous phases was a crucial factor determining emulsion stability, except for droplet size and viscosity of continuous phase. Applying this to Stokes’ law [42], the velocity of emulsion separation (*V*, m/s) is proportional to the density difference between dispersed and continuous phases (Δ*d* = *d*_1_ − *d*_2_, kg/m^3^), the viscosity of the continuous phase (*η*_2_, N s/m^2^), and the particle size of the dispersed phase (*r*, m) as follows:

(3) $$V=\frac{{2gr}^{2}{(d}_{1\;}{-\;d}_{2})}{{9\eta }_{2}}$$

Consequently, in addition to inhibiting Ostwald ripening and coalescence, which would lead to the formation of larger droplets, it is speculated that the density difference between the oil phase and the water phase is reduced. Thus, the stability of GEO emulsion is improved by overcoming gravity separation [17]. The densities of three VOs were listed as follows: CO (0.919 g/mL), SO (0.916 g/mL), and OO (0.909 g/mL). When GEO (density of 1.07 g/mL) was mixed with 10–20% VOs, its original high density could be modified to be closer to water (Δ*d* ≥ 0), and in turn rendered the stabilization of GEO emulsions. Nevertheless, when more than 33%—even 50%—VOs was added to GEO emulsions, it caused a negative density difference (Δ*d* < 0), thereby resulting in creaming and a less stable emulsion system [43]. It was worth noting that CO exhibited an effect of improving emulsion stability at ratios of 10%, 13%, and 20%, while SO at ratios of 13% and 20%, and OO only at 20% had a stabilizing effect on GEO emulsion, which contradicted the density order of VOs but was in agreement with the order of the chemical compatibility between GEO and VOs. Collectively, the results indicated that density difference had significant impact on GEO emulsion stability; meanwhile, the chemical compatibility between VOs and GEO also played an essential role in the emulsion stability, as evidenced by the droplet-size instability results of GEO emulsion in the presence of 13% OO and the difference in the volatile compounds of GEO-VO mixtures, especially for Diallyl disulfide and Diallyl trisulfide.

### 3.4. Morphologies and Encapsulation Efficiency of GEO Emulsions/Microcapsules

**Figure 1:**
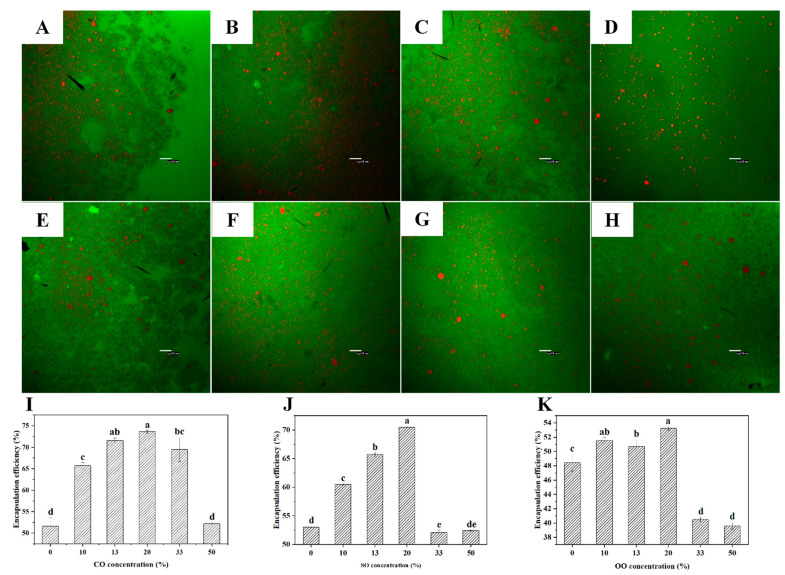
CLSM images of the freshly prepared GEO emulsions without VOs (**A**) and with the addition of 20% CO (**B**), 20% SO (**C**) and 20% OO (**D**) and the GEO emulsions after standing for 12 h without VOs (**E**) and with the addition of 20% CO (**F**), 20% SO (**G**) and 20% OO (**H**). The encapsulation efficiency of GEO microcapsules with different concentrations of CO (**I**), SO (**J**), and OO (**K**). Different letters between data indicate significant differences at *p* < 0.05.

According to the results of previous droplet-size distribution and TSI measurements, when the addition amount of each VO was 20%, correspondingly each GEO-VO emulsion had the highest stability. Therefore, CLSM images were obtained from the freshly prepared GEO emulsions and the emulsions after standing for 12 h in the presence of 20% VOs to evaluate the stability of the GEO emulsion further. As shown in Figure 1A–D, the oil droplets in the freshly prepared GEO and GEO-VO emulsions had relatively uniform and small droplet sizes, and were evenly dispersed, surrounding a network formed by WPC and IN. After standing for 12 h, different degrees of coalescence occurred in both GEO and GEO-VO emulsions (Figure 1E–H). Several large GEO droplets were formed in the pure GEO emulsion and GEO-VO emulsions, especially in the GEO-OO emulsion with 20% OO. No apparent aggregation appeared in the GEO emulsion with the addition of 20% CO after 12 h of standing. Moreover, compared to pure GEO emulsion, the GEO-CO emulsion displayed smaller droplet size and more uniform dispersion. According to Vall-llosera et al. [21], the CLSM images were analyzed using ImageJ software (Figure S3). As shown in Figure S3, the mean diameters of fresh emulsion droplets were 2.092 μm (GEO), 1.965 μm (GEO-CO), 1.922 μm (GEO-SO) and 2.339 μm (GEO-OO), respectively. After standing for 12 h, the mean diameters of GEO, GEO-CO, GEO-SO and GEO-OO emulsions changed to 2.611 μm, 2.417 μm, 2.507 μm and 3.222 μm, respectively. The results indicated that 20% of CO had a certain inhibitory effect on the formation of large particles and promoted the stability of GEO emulsion, which agreed with the results obtained from the laser analyzer. These observations were reminiscent of the previous elaboration on Ostwald ripening, which suggested the incompatibility of GEO chemical components and OO resulted in the emulsion being unstable with a significant increase in the droplet size and even oil–water separation [17]. As can be seen in Table 1, OO (46.91%) was inferior to CO (43.47%) and SO (43.15%), at least for the retention of Diallyl disulfide.

Microencapsulation by spray drying is widely employed in the production of dry and stable flavor-containing additives [44]. Improving encapsulation efficiency and flavor retention is critical to the quality of microcapsules. In Figure 1I–K, the EE value increased in GEO microcapsules in the concentration range of 0–20% VOs, but dramatically diminished by adding 50% CO and 33–50% SO and OO. As demonstrated by Silva et al. [43], emulsion stability was an essential parameter in influencing the process of spray drying; thus, the fact that low EE values were observed in the GEO-VO microcapsules with the addition of 50% CO, 33–50% SO and OO, correspondingly, can be attributed to the instability of GEO-VO emulsions with high TSI values. Fernandes et al. [13,14] reported that when WPC and IN were employed as wall materials for spray-drying microencapsulation, the rosemary essential oil and ginger essential oil had EE values of 37.7% and 48.14%, respectively. In this study, when 20% of VOs were added, the highest EE values of GEO-CO, GEO-SO, and GEO-OO microcapsules were 73.65%, 70.5%, and 53.24%, respectively. It indicated that adding VOs, especially for CO and SO, could enhance the encapsulation efficiency of GEO compared to that of pure GEO microcapsules (approximately 50%) stabilized by WPC and IN.

**Figure 2:**
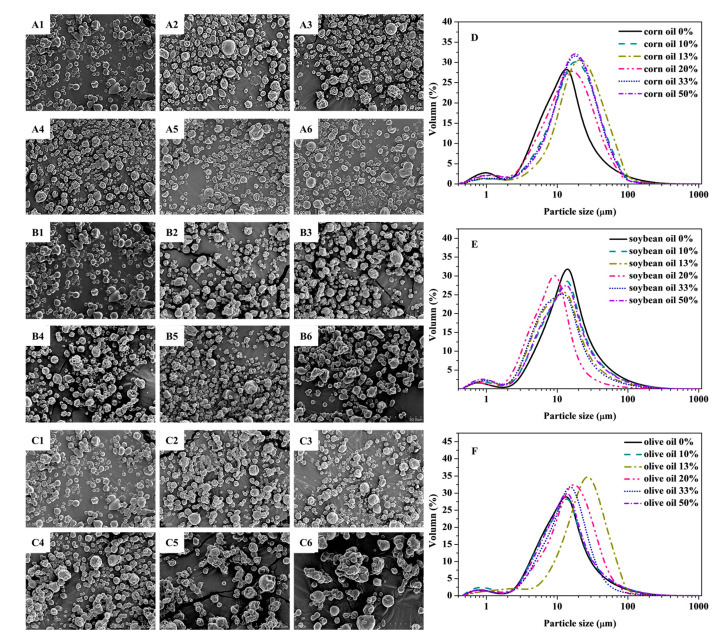
SEM images of GEO microcapsules prepared with different concentrations (0%, 10%, 13%, 20%, 33% and 50%) of CO (**A1**–**A6**), SO (**B1**–**B6**) and OO (**C1**–**C6**). The particle size distribution of GEO microcapsules with different concentrations of CO (**D**), SO (**E**) and OO (**F**).

The encapsulation effect of GEO was further evaluated by using scanning electron microscopy (SEM) and particle-size distribution measurements. Figure 2A–C showed the SEM images of the GEO microcapsules prepared with different concentrations (0–50%) of CO, SO, and OO. The SEM revealed the presence of VOs enhancing the microencapsulation efficiency of GEO. The GEO and GEO-VO microcapsules exhibited spherical and oval-shaped particles with irregular surfaces and concavities typically formed upon the spray-drying process, owing to the uneven shrinkage of the particles caused by the rapid evaporation of the liquid drops at the initial stage of spray-drying [45,46]. With the addition of 20% VOs, most of the GEO-VO microcapsules were dispersed, and the surface was smooth and complete without apparent cracks. Compared with pure GEO microcapsules, the medium particle size of GEO-CO microcapsules was slightly increased to about 11 μm (Figure 2D), which revealed larger particle size manifesting higher encapsulation efficiency. However, GEO microcapsules displayed varying degrees of aggregation when mixed with the excess VOs, especially for 50% SO and 33–50% OO. The different performance of three VOs on the encapsulation efficiency or the retention of volatile constituents can be attributed to the changes in the interactions between GEO chemical compositions and wall materials [14], as revealed by Fourier transform infrared spectra (FTIR). Figure S5 showed that GEO bands at 990 cm^−1^ were associated with C–S stretching [16]. WPC had absorption bands at 1515 and 1630 cm^−1^, which resulted from C=O stretching belonging to amide I bond. IN showed a strong band at 1017 cm^−1^ assigned to C–O–C vibration mode [14]. The FTIR result indicated that the C–S bonds were weakened due to the microencapsulation of GEO. When 20% VOs were mixed with GEO, the FTIR absorption band at 1040 cm^−1^ had its intensity reduced due to the high encapsulation efficiency of GEO, affecting the interactions between WPC and IN. Furthermore, DSC curves revealed that the thermal stability of GEO was significantly improved after microencapsulation. Although the weight loss rate of three VOs was distinct, their addition did not affect the thermal stability of GEO microcapsules (Figure S6).

### 3.5. Controlled Release

**Figure 3:**
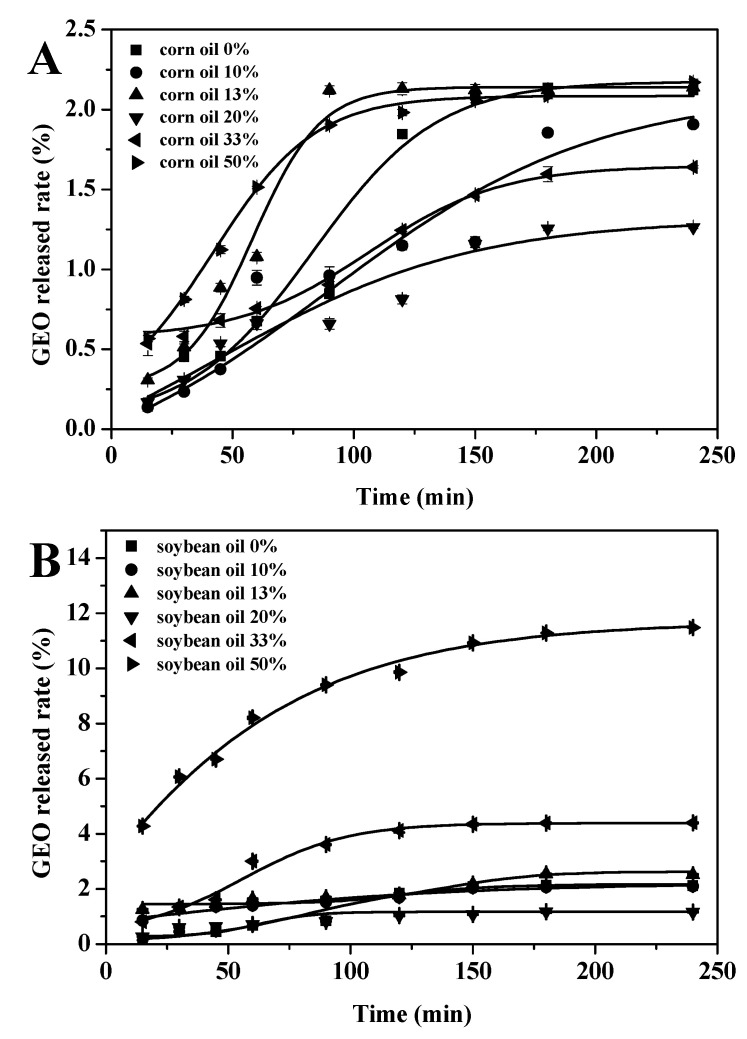

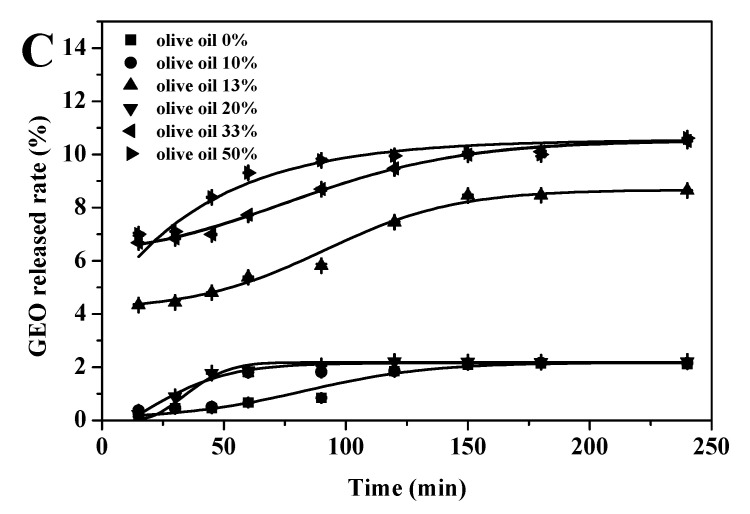
GEO released curves of microcapsules prepared with different concentrations of CO (**A**), SO (**B**) and OO (**C**).

The release properties of the GEO and GEO-VO microcapsules were determined as a function of time (Figure 3). The stability of the emulsion and droplet size have important effects on the sustained release and shelf life of the volatile compounds in the microencapsulated oils [47], and the encapsulation affects positively the stability of bioactive molecules [48]. Consistent with the results of the size distribution and the physical stability, the GEO-CO microcapsules with CO concentrations of 10%, 13% and especially for 20% showed a slower GEO release than those pure GEO microcapsules (Figure 3A). The GEO was released faster in the first hour of incubation and then was gradually decreased. Microencapsulation has important effects on the release properties of the enclosed contents. Zhang et al. [49] prepared fragrance microcapsules encapsulated by maltodextrin and resistant starch as wall materials and found its release rate was mild and gentle. Ozdemir et al. [50] used gum Arabic, maltodextrin and whey protein isolate as the wall materials to gain basil essential oil microcapsules, with a release rate of 58.97%. The rapid release of GEO in the initial stage might be due to the swollen hydrated wall materials leading to an increase in the permeability of the wall to the solvent and to a certain extent the release of the adsorbed GEO at microcapsules surface into the media [51,52]. A similar trend was observed in the release behavior of GEO-SO microcapsules, and the release rate of the encapsulated GEO with the addition of 20% SO was well-controlled. Nevertheless, the excess addition of 33–50% CO or SO could reduce the encapsulation efficiency, thus increasing the amounts of GEO at the surface and thereby raising the released rate of the GEO. Unfortunately, the release rate of GEO-OO microcapsules was higher than that of pure GEO microcapsules, which probably was due to the unstable nature of olive oil itself and the incompatibility of GEO and olive oil, which was not conducive to the formation of polymer networks controlling the GEO release. In addition, the release rate curve of each GEO microcapsule gradually flattened with time, indicating that the GEO was successfully embedded and slowly released.

### 3.6. Antibacterial Activity

**Figure 4:**
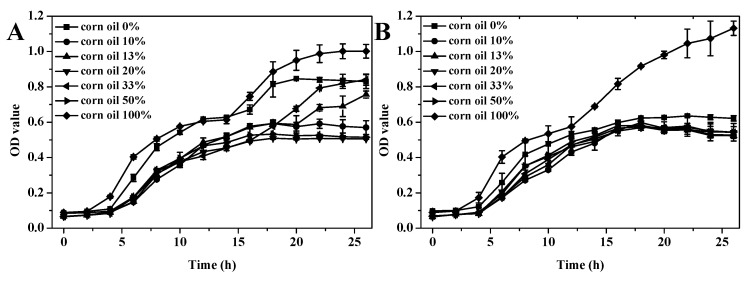

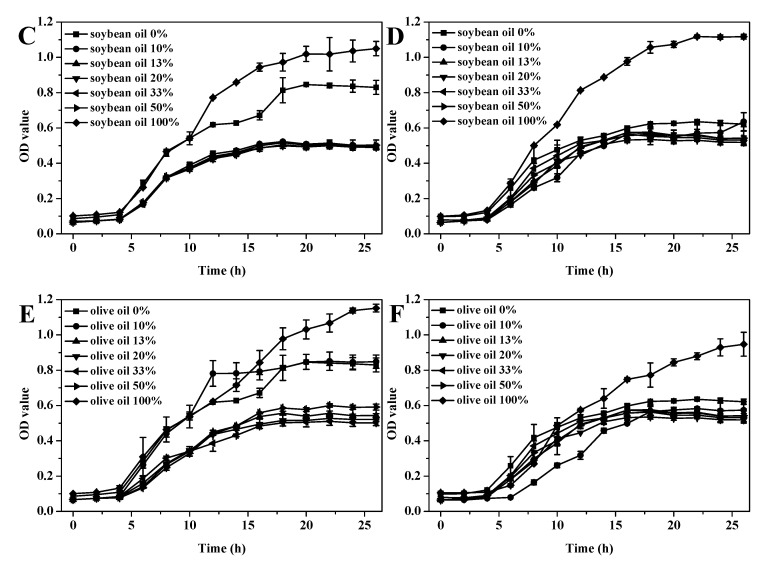
Growth curves of *E. coli* treated by GEO-CO (**A**), GEO-SO (**C**) and GEO-OO (**E**) microcapsules and *S. aureus* treated by GEO-CO (**B**), GEO-SO (**D**) and GEO-OO (**F**).

Growth curves of Gram-negative bacteria *E. coli* and Gram-positive bacteria *S. aureus* treated by GEO-VO microcapsules with different concentrations of VOs were shown in Figure 4. For the control group of pure VO-containing microcapsules without GEO addition, the growth curves of both *E. coli* and *S. aureus* reached a stable growth stage in 20 h, and the absorbance value was approximately at 1.0. A reduction of both *E. coli* and *S. aureus* growth was observed for all GEO-VO microcapsules, with the 20% CO-containing GEO microcapsules demonstrating the highest inhibition effectiveness. The stable growth stages for both *E. coli* and *S. aureus* were reached in 16, 17 and 17 h with the minimum absorbance value in the range of 0.5–0.6 when treated with 20% CO, SO and OO, respectively, indicating that GEO-VOs exhibited good inhibition effectiveness in the growth of both *E. coli* and *S. aureus* in an encapsulation efficiency-dependent behavior, which was associated with the physical stability of the GEO emulsion and the volatile compounds of GEO. Other researchers also found that the garlic essential oil was particularly efficient and showed activity on a large panel of pathogens [53]. As reported by Casella et al. [54], the main antibacterial constituents in GEO were allyl-group-containing compounds, including Diallyl sulfide, Diallyl disulfide, Diallyl trisulfide, and Diallyl tetrasulfide. After GEO was mixed with different VOs, the compatibility in the chemical composition of GEO and VOs should also be considered except for the influencing factors for GEO emulsions and microcapsules themselves.

## 4. Conclusions

The data indicate that garlic essential oil–vegetable oil (GEO-VO) emulsions, especially for GEO-CO (corn oil) emulsions, are more stable than pure GEO emulsions, as demonstrated by droplet-size distribution, Turbiscan stability index measurements, and confocal laser scanning microscopy. Therefore, the GEO-CO microcapsules showed the highest encapsulation efficiency among all GEO microcapsules with controlled release and effective antibacterial activity on both *Escherichia coli* and *Staphylococcus aureus*. VOs, especially for 20% CO, enhance the stability of GEO emulsions and the encapsulation efficiency of GEO microcapsules, presumably by (1) reducing the density difference between oil and water phases to protect from gravitational separation and by (2) improving the chemical compatibility of GEO and Vos, and thus to a certain extent inhibiting Ostwald ripening. Nevertheless, further studies are needed to fully understand the effect of VOs on the GEO emulsion and microcapsules in other aspects of physicochemical properties of VOs, such as viscosity, conductivity, and refining degree.

## Figures and Tables

**Table 1:** Volatile compounds of GEO and GEO-VO mixtures.

GEO		GEO-CO Mixture		GEO-SO Mixture		GEO-OO Mixture	
Compounds	Percentage (%)	Compounds	Percentage (%)	Compounds	Percentage (%)	Compounds	Percentage (%)
Ketene	0.36	Ketene	1.02	Ketene	1.00	Ketene	0.72
3-prop-2-enylsulfanylprop-1-ene *	6.20	3-prop-2-enylsulfanylprop-1-ene	8.12	3-prop-2-enylsulfanylprop-1-ene	8.06	3-prop-2-enylsulfanylprop-1-ene	7.08
3*H*-dithiole *	4.21	3*H*-dithiole	2.13	3*H*-dithiole	2.86	3*H*-dithiole	1.86
(4*R*)-1-methyl-4-prop-1-en-2-ylcyclohexene	1.56	(4*R*)-1-methyl-4-prop-1-en-2-ylcyclohexene	1.11	(4*R*)-1-methyl-4-prop-1-en-2-ylcyclohexene	1.10	(4*R*)-1-methyl-4-prop-1-en-2-ylcyclohexene	1.10
3-(prop-2-enyldisulfanyl)prop-1-ene *	30.34	3-prop-2-enylsulfanylprop-1-ene	43.47	3-prop-2-enylsulfanylprop-1-ene	43.15	3-prop-2-enylsulfanylprop-1-ene	46.91
3-ethenyl-3,6-dihydrodithiine *	4.80	3-ethenyl-3,6-dihydrodithiine	3.02	3-ethenyl-3,6-dihydrodithiine	3.00	3-ethenyl-3,6-dihydrodithiine	2.88
4H-trithiine *	5.70	4*H*-trithiine	4.60	4*H*-trithiine	4.74	4*H*-trithiine	4.56
2-ethenyl-4*H*-1,3-dithiine *	6.49	2-ethenyl-4*H*-1,3-dithiine	5.79	2-ethenyl-4*H*-1,3-dithiine	5.75	2-ethenyl-4*H*-1,3-dithiine	5.70
1-methoxy-4-[(*E*)-prop-1-enyl]benzene	0.37	3-(prop-2-enyltrisulfanyl)prop-1-ene	27.13	3-(prop-2-enyltrisulfanyl)prop-1-ene	26.93	3-(prop-2-enyltrisulfanyl)prop-1-ene	25.64
3-(prop-2-enyltrisulfanyl)prop-1-ene *	28.18	5-methyltetrathiane	1.18	5-methyltetrathiane	1.17	5-methyltetrathiane	1.21
5-methyltetrathiane	2.86	3-(prop-2-enyltetrasulfanyl)prop-1-ene	2.27	3-(prop-2-enyltetrasulfanyl)prop-1-ene	2.25	3-(prop-2-enyltetrasulfanyl)prop-1-ene	2.31
3,3,7-trimethyl-8-methylidenetricyclo[5.4.0.02,9]undecane	1.30						
2,6-ditert-butyl-4-methylphenol	0.31						
3-(prop-2-enyltetrasulfanyl)prop-1-ene *	3.88						
1-(3,5-ditert-butyl-4-hydroxyphenyl)propan-1-one	3.04						
2-prop-2-enylsulfanyl-1-(prop-2-enyltrisulfanyl)propane *	0.38						
Total compounds	99.98		99.84		100.00		99.97
Sulfur-containing compounds	90.18		96.53		96.74		96.94
Diallyl disulfide and Diallyl trisulfide	58.52		70.60		70.08		72.55

Notes: * Main sulfur-containing compounds in GEO; 3-prop-2-enylsulfanylprop-1-ene: Diallyl sulfide; 3-(prop-2-enyldisulfanyl)prop-1-ene: Diallyl disulfide; 3-(prop-2-enyltrisulfanyl)prop-1-ene: Diallyl trisulfide; 3-(prop-2-enyltetrasulfanyl)prop-1-ene: Diallyl tetrasulfide; 2-prop-2-enylsulfanyl-1-(prop-2-enyltrisulfanyl) propane: Trisulfide, 2-propenyl 2-(2-propenylthio)propyl.

**Table 2:** Droplet-size distribution, polydispersity index and Turbiscan stability index of GEO and GEO-VO emulsions.

VO Concentration	Mean Droplet Size (μm)		Polydispersity Index		Turbiscan Stability Index			
	0 h	12 h	0 h	12 h	2 h	4 h	6 h	12 h
0%	1.31 ± 0.01 ^e^	2.25 ± 0.03 ^c^	3.27 ± 0.01 ^a^	3.31 ± 0.02 ^ab^	0.06 ± 0.00 ^b^	0.10 ± 0.00 ^c^	0.18 ± 0.00 ^b^	0.68 ± 0.01 ^b^
10%	1.54 ± 0.03 ^d^	2.00 ± 0.01 ^d^	2.83 ± 0.02 ^d^	3.12 ± 0.13 ^b^	0.04 ± 0.00 ^d^	0.08 ± 0.00 ^d^	0.13 ± 0.00 ^c^	0.39 ± 0.02 ^d^
13%	1.61 ± 0.02 ^c^	2.06 ± 0.03 ^d^	3.07 ± 0.04 ^b^	3.06 ± 0.01 ^b^	0.03 ± 0.00 ^e^	0.06 ± 0.00 ^e^	0.14 ± 0.00 ^c^	0.36 ± 0.01 ^d^
20%	1.25 ± 0.02 ^f^	1.95 ± 0.01 ^d^	3.31 ± 0.04 ^a^	2.67 ± 0.03 ^c^	0.02 ± 0.00 ^f^	0.03 ± 0.00 ^f^	0.05 ± 0.00 ^d^	0.08 ± 0.00 ^e^
33%	2.13 ± 0.01 ^b^	3.17 ± 0.14 ^b^	2.96 ± 0.01 ^c^	3.42 ± 0.14 ^a^	0.05 ± 0.00 ^c^	0.15 ± 0.00 ^b^	0.19 ± 0.00 ^b^	0.51 ± 0.02 ^c^
50%	2.54 ± 0.01 ^a^	3.61 ± 0.00 ^a^	3.08 ± 0.03 ^b^	3.59 ± 0.12 ^a^	0.15 ± 0.00 ^a^	0.24 ± 0.00 ^a^	0.41 ± 0.02 ^a^	2.07 ± 0.10 ^a^
0%	1.31 ± 0.01 ^d^	2.25 ± 0.03 ^c^	3.27 ± 0.01 ^a^	3.31 ± 0.02 ^b^	0.06 ± 0.00 ^d^	0.10 ± 0.00 ^c^	0.18 ± 0.00 ^d^	0.68 ± 0.01 ^b^
10%	1.95 ± 0.01 ^c^	3.06 ± 0.04 ^b^	2.83 ± 0.02 ^c^	3.14 ± 0.04 ^b^	0.13 ± 0.01 ^b^	0.27 ± 0.01 ^a^	0.41 ± 0.01 ^b^	0.66 ± 0.04 ^b^
13%	2.15 ± 0.14 ^b^	2.27 ± 0.06 ^c^	3.37 ± 0.14 ^a^	3.12 ± 0.07 ^b^	0.06 ± 0.00 ^d^	0.11 ± 0.00 ^c^	0.16 ± 0.00 ^e^	0.35 ± 0.01 ^c^
20%	1.28 ± 0.02 ^d^	2.07 ± 0.01 ^c^	3.07 ± 0.04 ^b^	3.04 ± 0.16 ^b^	0.02 ± 0.00 ^e^	0.03 ± 0.00 ^d^	0.04 ± 0.00 ^f^	0.15 ± 0.01 ^d^
33%	2.12 ± 0.01 ^b^	3.25 ± 0.03 ^b^	2.96 ± 0.01 ^bc^	3.36 ± 0.13 ^b^	0.09 ± 0.00 ^c^	0.16 ± 0.00 ^b^	0.23 ± 0.00 ^c^	0.60 ± 0.00 ^b^
50%	2.54 ± 0.01 ^a^	5.64 ± 0.35 ^a^	3.08 ± 0.03 ^b^	4.43 ± 0.21 ^a^	0.16 ± 0.00 ^a^	0.26 ± 0.01 ^a^	0.53 ± 0.00 ^a^	2.17 ± 0.13 ^a^
0%	1.31 ± 0.01 ^c^	2.25 ± 0.03 ^e^	3.27 ± 0.01 ^a^	3.31 ± 0.02 ^b^	0.06 ± 0.00 ^c^	0.10 ± 0.00 ^d^	0.18 ± 0.00 ^d^	0.68 ± 0.00 ^b^
10%	1.20 ± 0.00 ^d^	5.12 ± 0.22 ^c^	2.61 ± 0.02 ^c^	3.27 ± 0.01 ^b^	0.16 ± 0.00 ^b^	0.11 ± 0.00 ^cd^	0.15 ± 0.00 ^e^	0.41 ± 0.02 ^c^
13%	1.20 ± 0.00 ^d^	4.23 ± 0.12 ^d^	2.51 ± 0.05 ^d^	3.31 ± 0.14 ^b^	0.17 ± 0.01 ^b^	0.34 ± 0.01 ^b^	0.54 ± 0.01 ^b^	0.69 ± 0.02 ^b^
20%	1.37 ± 0.04 ^c^	3.87 ± 0.00 ^d^	2.31 ± 0.03 ^e^	3.04 ± 0.01 ^c^	0.03 ± 0.00 ^d^	0.06 ± 0.00 ^e^	0.11 ± 0.00 ^f^	0.20 ± 0.00 ^d^
33%	1.82 ± 0.01 ^b^	6.95 ± 0.37 ^b^	2.88 ± 0.03 ^b^	3.15 ± 0.01 ^bc^	0.07 ± 0.00 ^c^	0.12 ± 0.01 ^c^	0.25 ± 0.01 ^c^	0.69 ± 0.02 ^b^
50%	3.24 ± 0.04 ^a^	9.94 ± 0.35 ^a^	2.58 ± 0.03 ^cd^	3.71 ± 0.05 ^a^	0.33 ± 0.01 ^a^	0.55 ± 0.00 ^a^	0.77 ± 0.00 ^a^	2.86 ± 0.00 ^a^

Note: Data are expressed as mean ± standard deviation. Different letters between data for each GEO-VO emulsion indicate significant differences at *p* < 0.05.

## Supplementary Materials

The following are available online at https://www.mdpi.com/article/10.3390/foods10071637/s1. Figure S1: TSI curves of GEO emulsions stabilized by different types and ratios of wall materials. Figure S2: Variations in the profiles of the majority compounds in GEO emulsions stabilized by different types and ratios of wall materials. Figure S3: The size distribution analysis for GEO (A), GEO-CO (B), GEO-SO (C) and GEO-OO (D) emulsions and the emulsions after standing for 12 h (E, F, G, H), obtained by ImageJ software analysis based on the images confocal laser scanning microscopy of laser results. Figure S4: Droplet size distributions of the freshly prepared GEO and GEO-VO emulsions (A, C and E) and the emulsions after standing for 12 h (B, D and F). Figure S5: FTIR spectra of GEO and GEO-VO microcapsules. Figure S6: TGA curves of GEO and GEO-VO microcapsules. Table S1: The experimental design for preparing GEO and GEO-VO emulsions. Table S2: Volatile compounds of corn oil, soybean oil and olive oil.
