# Supplementary material for: Mixing Oil-Based Microencapsulation of Garlic Essential Oil: Impact of Incorporating Three Commercial Vegetable Oils on the Stability of Emulsions
Source: Foods. 2021 Jul 15;10(7):1637. doi: 10.3390/foods10071637 (PMC8305996; doi:10.3390/foods10071637)
Supplement: Supplementary file 1 [file foods-10-01637-s001.zip › foods-1257799-supplementary.pdf]

SUPPORTING INFORMATION

Mixing oil-based Microencapsulation of Garlic Essential Oil: Impact of Incorporating Three Commercial Vegetable Oils on the Stability of Emulsions

Yunjiao Zhao[1], Rui Liu[1,*], Cuiping Qi[1], Wen Li[1], Mohamed Rifky[1], Min Zhang[2,*], Ping Xiao[3], Tao Wu[1], Wenjie Sui[1]

[1]State Key Laboratory of Food Nutrition and Safety, Tianjin University of Science & Technology, Tianjin, 300457, China

[2]Tianjin Agricultural University, Tianjin, 300384, P.R. China

[3]Tianjin Chunfa Bio-Technology Group Co., Ltd, Tianjin, 300300, P.R. China

Correspondence: lr@tust.edu.cn; zm0102@sina.com; xiaoping19860724@126.com

Preparation of GEO and GEO-VO emulsions

The formulation of wall materials (WPC/IN and WPC/MD) was optimized in terms of emulsion physical and flavor stability according to previous studies [1,2]. The experiment design was set as shown in Table S1 and the stability and flavoring retention results were shown in Fig. S1 and Fig. S2, respectively.

Table S1. The experimental design for preparing GEO and GEO-VO emulsions.

GEO emulsions (g/30g)					Emulsification methods	
WPC	IN	MD	VO	GEO	Homogenization	Sonication
4.5	1.5	-	0.3	1.5	√	√
4.0	2.0	-	0.3	1.5	√	√
3.0	3.0	-	0.3	1.5	√	√
2.0	4.0	-	0.3	1.5	√	√
1.5	4.5	-	0.3	1.5	√	√
4.5	-	1.5	0.3	1.5	√	√
4.0	-	2.0	0.3	1.5	√	√
3.0	-	3.0	0.3	1.5	√	√
2.0	-	4.0	0.3	1.5	√	√
1.5	-	4.5	0.3	1.5	√	√

Fig. S1 TSI curves of GEO emulsions stabilized by different types and ratios of wall materials.

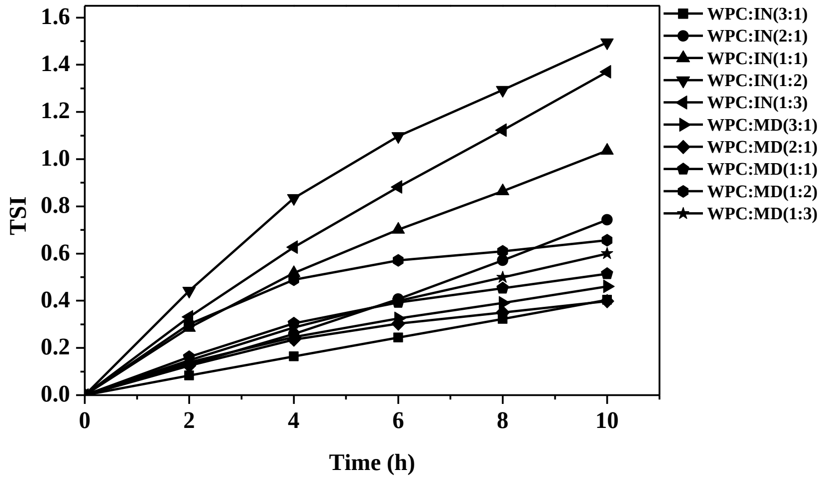

Fig. S2 Variations in the profiles of the majority compounds in GEO emulsions

stabilized by different types and ratios of wall materials.

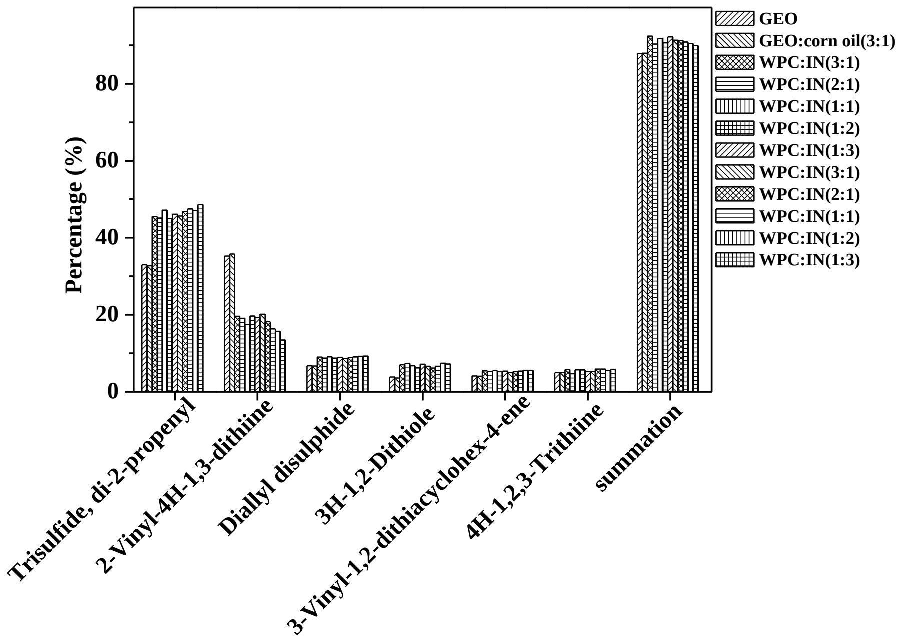

GEO emulsion had the highest physical stability and best flavor retention capacity when emulsified and stabilized by WPC and IN at a ratio of 3:1 as wall materials. Therefore, WPC:IN at the ratio of 3:1 was further employed to study the effect of VOs addition on the stability of GEO emulsions and encapsulation efficiency of GEO microcapsules.

Table S2 Volatile compounds of corn oil, soybean oil and olive oil.

**Corn oil**		**Soybean oil**		**Olive oil**			
Compounds	Percentage (%)	Compounds	Percentage (%)	Compounds	Percentage (%)	Compounds	Percentage (%)
(2*R*)-2-amino-3-sulfinopropanoic acid	3.16	(2*R*)-2-amino-3-sulfinopropanoic acid	8.07	(2*R*)-2-amino-3-sulfinopropanoic acid	0.45	3,4-bis (methoxycarbonyl) benzoic acid	6.71
2-(methylamino) ethanesulfonic acid	29.27	2-(methylamino)ethanesulfonic acid	12.27	2-(methylamino) ethanesulfonic acid	1.52	(*Z*)-1-methoxyhex-3-ene	2.21
Urea	9.28	Butan-2-amines	1.55	Formamide	6.39	(*E*)-hex-2-enal	5.23
2-aminopropan-1-ol	6.95	Acetic acid	8.70	2,3-dimethyloxirane	0.87	(*E*)-hex-3-en-1-ol	8.57
3-methylbutanal	2.68	Hexane	8.44	Acetic acid	5.75	6-phenylmethoxyhexoxymethylbenzene	3.97
Pentanal	21.93	2-fluoroacetamide	17.88	Ethyl Acetate	2.23	3-methyl-2-phenylpyrazolo [1,5-a] pyridine	0.91
Hexanal	26.72	Toluene	3.47	Pentanal	0.32		
		Propylcyclopentane	15.72	Cyclopentanol	0.35	(5*E*)-3-ethylocta-1,5-diene	1.18

		Hexanal	22.06	3-methylbutanal	1.74	3-*N*-(1,3,5-trimethylpyrazol-4-yl)-1*H*-1,2,4-triazole-3,5-diamine	1.93
		(2,4-*ditert*-butylphenyl) 5-hydroxypentanoate	1.84	Ethyl *N*-propan-2-yloxycarbamate	0.27	Tris (trimethylsilyl) arsorite	0.35
				(*E*)-2-methylbut-2-enal	0.45	(4-cyanophenyl) 4-(2-propoxyethoxy) benzoate	0.94
				Toluene	0.66	Ethylidenecyclohexane	2.28
				(*Z*)-pent-2-en-1-ol	0.40	[(*Z*)-hex-3-enyl] acetate	16.87
				(2*R*)-2-aminopropanoic acid	0.31	2,6,6-trimethylbicyclo [3.1.1] hept-2-ene	1.23
				Pentylcyclopropane	0.24	Nonanal	1.63
				2,4-dimethylhexane	17.84	Ethyl *N*-(1-adamantylcarbamothioyl) carbamate	1.31
				Oct-1-ene	0.21	Undecan-1-ol	3.18
				Cyclooctane	0.20	(1*R*,4*R*,4*aS*)-4,7-dimethyl-1-propan-2-yl-1,2,3,4,4*a*,5-hexahydronaphthalene	1.29
**Total compounds**	99.99		100.00				99.99
**Sulfur-containing compounds**	32.43		20.34				1.97

**Diallyl disulfide and Diallyl trisulfide**	0.00	0.00		0.00

Fig. S3 The size distribution analysis for GEO (A), GEO-CO (B), GEO-SO (C) and GEO-OO (D) emulsions and the emulsions after standing for 12 h (E, F, G, H), obtained by ImageJ software analysis based on the images confocal laser scanning microscopy of laser results.

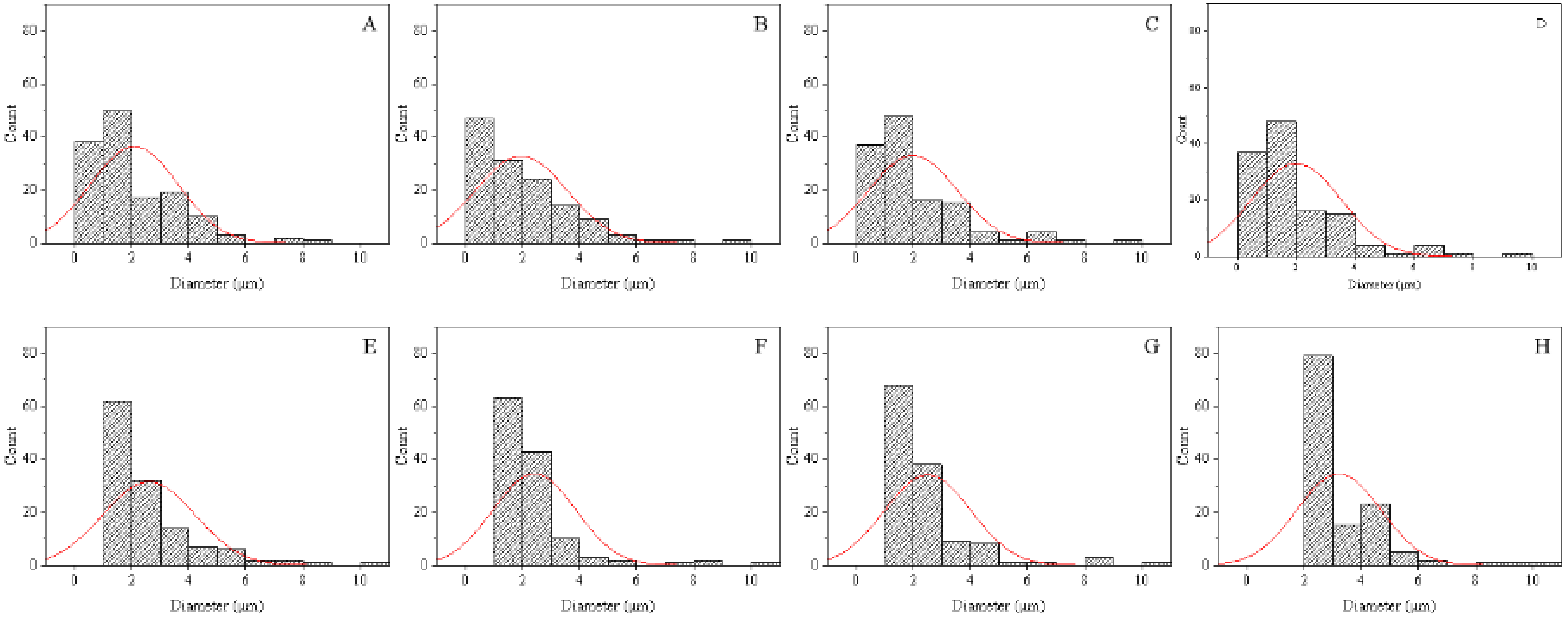

Fig. S4 Droplet size distributions of the freshly prepared GEO and GEO-VO emulsions (A, C and E) and the emulsions after standing for 12 h (B, D and F).

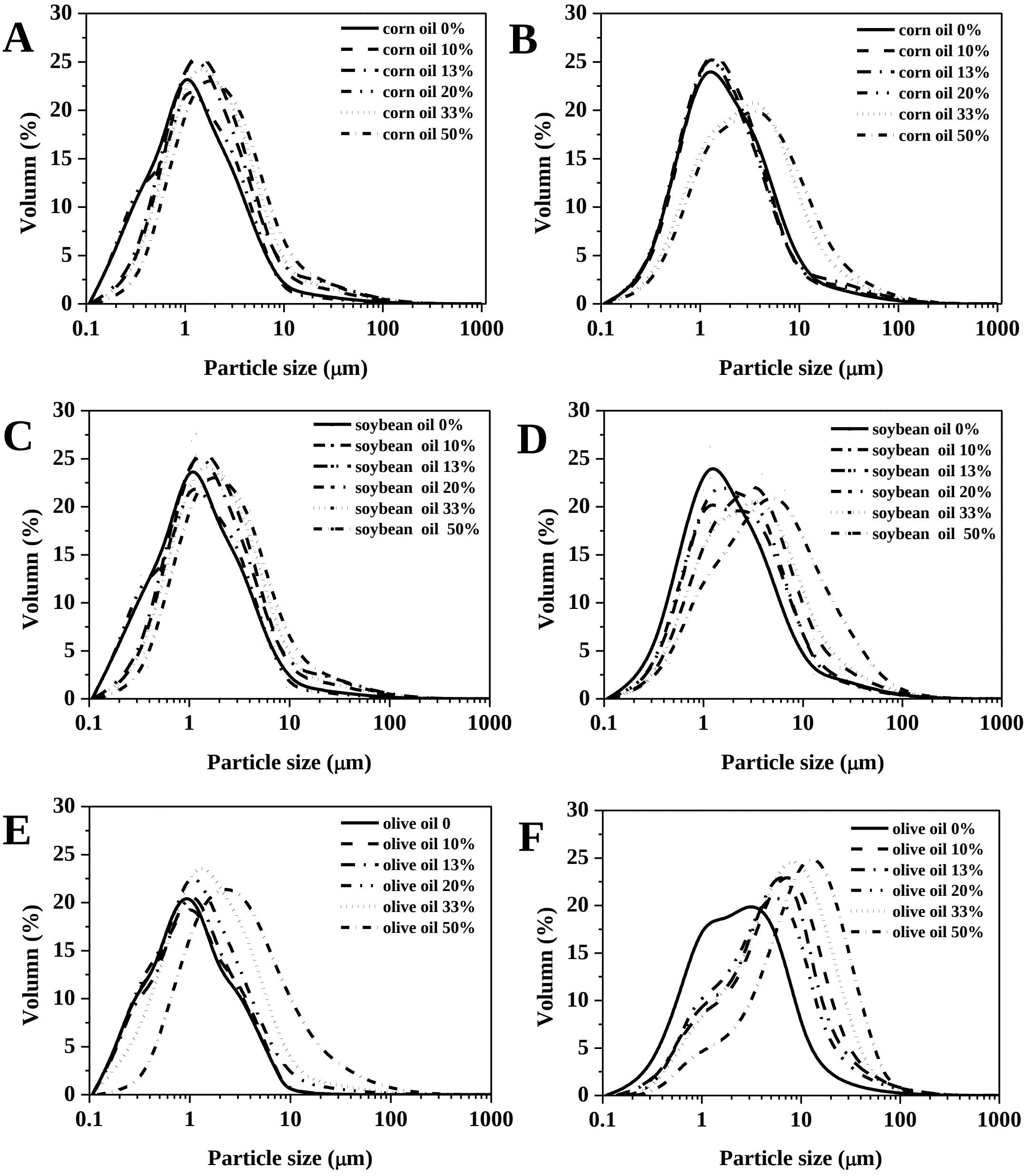

Fig. S5 FTIR spectra of GEO and GEO-VO microcapsules.

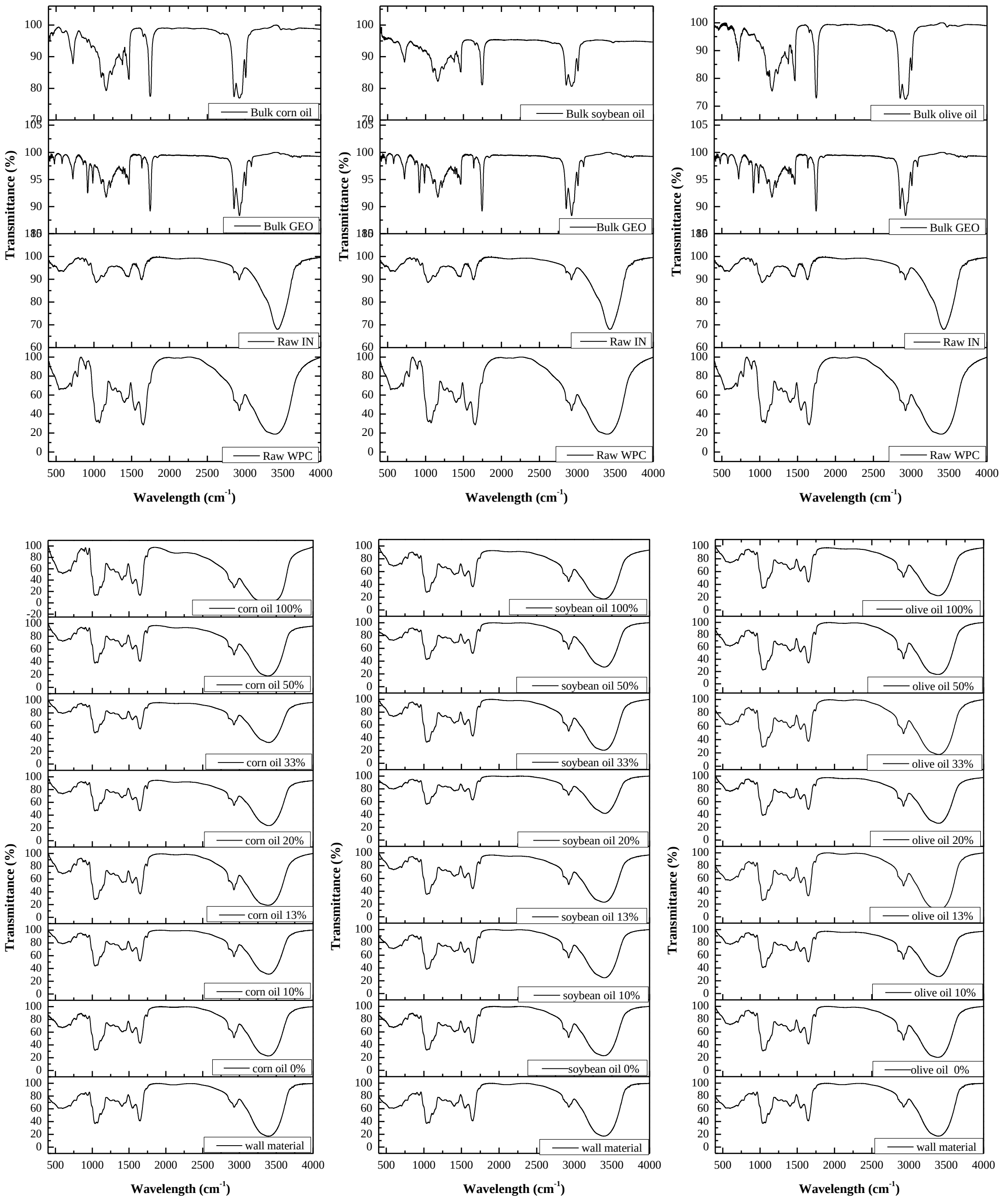

Fig. S6 TGA curves of GEO and GEO-VO microcapsules.

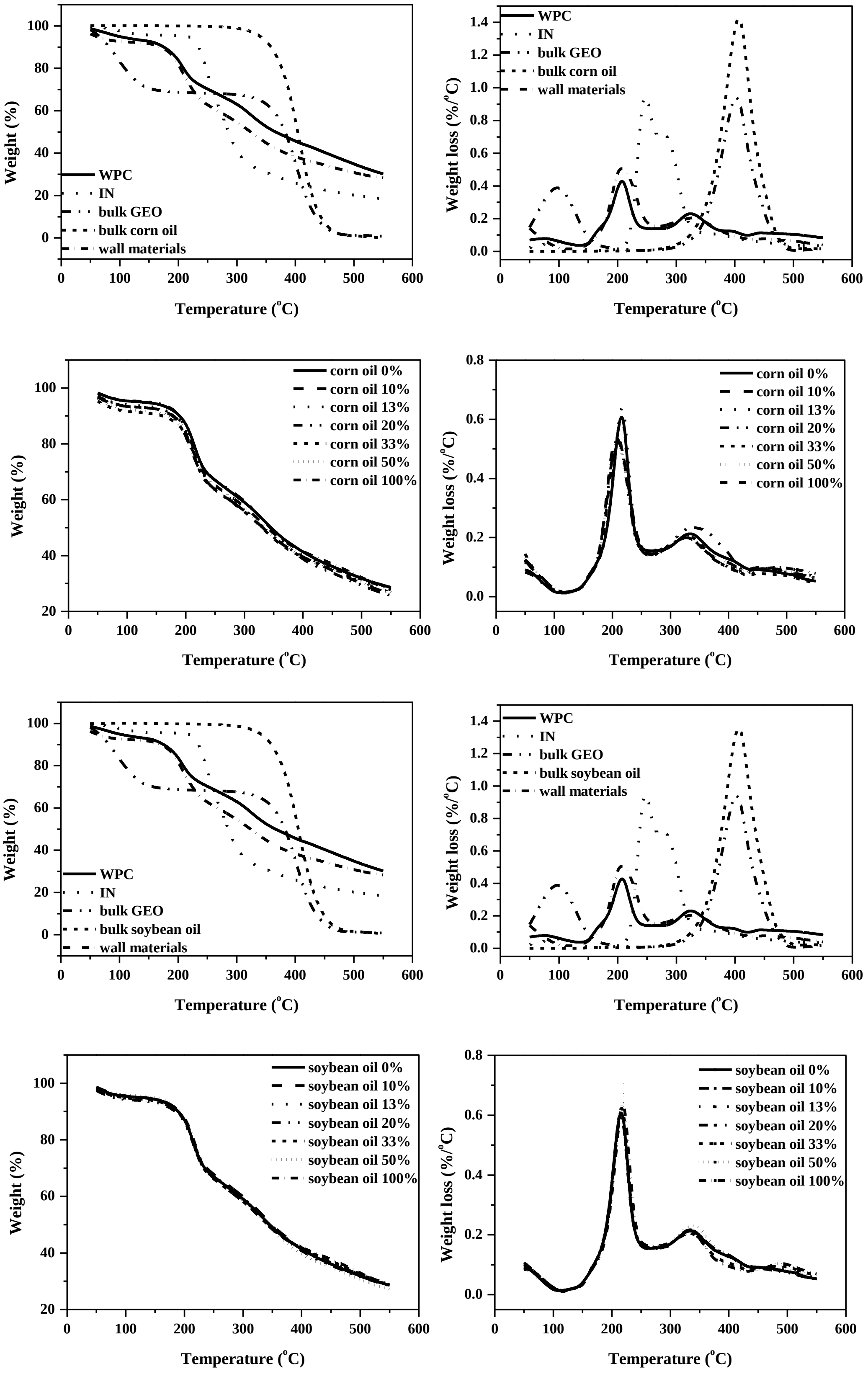

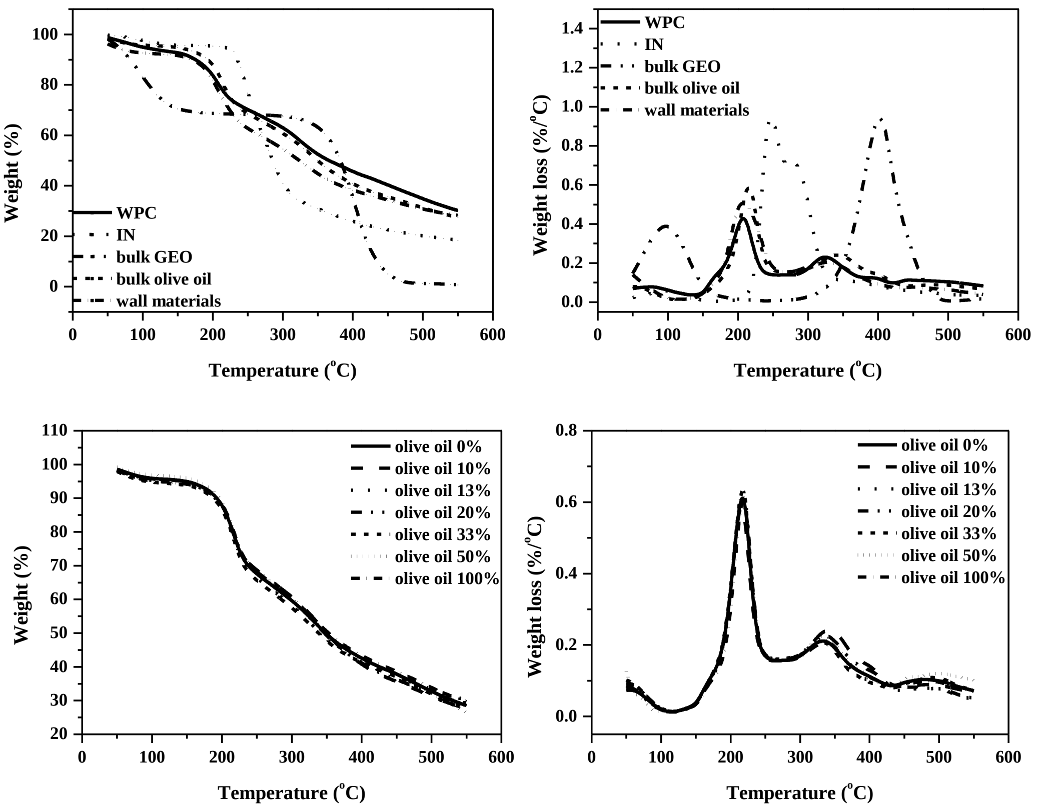
WPC
IN
bulk GEO
bulk olive oil
wall materials
Weight (%)
Temperature (°C)
Weight loss (%/°C)
olive oil 0%
olive oil 10%
olive oil 13%
olive oil 20%
olive oil 33%
olive oil 50%
olive oil 100%

References

1. Fernandes, R.V.D.; Borges, S.V.; Botrel, D.A. Gum arabicistarchimaltodextrin/inulin as wall materials on the microencapsulation of rosemary essential oil. Carbohydrate Polymers 2014, 101, 524-532, doi:10.1016/j.carbpol.2013.09.083.
2. Fernandes, R.V.D.; Silva, E.K.; Borges, S.V.; de Oliveira, C.R.; Yoshida, M.I.; da Silva, Y.F.; do Carmo, E.L.; Azevedo, V.M.; Botrel, D.A. Proposing Novel Encapsulating Matrices for Spray-Dried Ginger Essential Oil from the Whey Protein Isolate-Inulin/Maltodextrin Blends. Food and Bioprocess Technology 2017, 10, 115-130, doi:10.1007/s11947-016-1803-1.
